# Variations in epidermal trichomes of a mystic weed *Parthenium hysterophorus* L. from semi-arid regions of Barmer, Rajasthan (India)

**DOI:** 10.3389/fpls.2024.1363774

**Published:** 2024-03-05

**Authors:** Vimala, Dinesh Hans

**Affiliations:** ^1^ Department of Botany, Mankaya Lal Verma Government College, Bhilwara, Rajasthan, India; ^2^ Department of Botany, Seth Ranglal Kothari Government College, Rajsamand, Rajasthan, India

**Keywords:** Asteraceae, Barmer, capitulum, Parthenium hysterophorus L., trichomes

## Abstract

The Asteraceae family of plants, which has 16,000–17,000 genera and 24,000–30000 species, is diverse and widely spread, notably in the tropics and subtropics. Asteraceae has capitula head bracket traits that are unique to this genus of plants. This study’s goal was to identify the micro-morphological makeup of the trichomes in *Parthenium hysterophorus* L. Invasive weed *Parthenium hysterophorus* L. is erect, short-lived fast-growing plant is found in hot areas and is known for its luxuriant growth. As the stem attains maturity, becomes harder. Mature stems are greenish and coated in tiny, soft hairs called hirustles. Later leaves are simple and deeply pinnatifid, while early leaves create a rosette habitat. Hundreds of tiny flower heads, or capitulum, are arranged in clusters at the apex of the branches. Trichomes are epidermal appendages that are frequently seen on the leaves, stems, and fruits of plants. There are two types of trichomes: glandular and non-glandular. The immature leaves and stem of Parthenium hysterophorus L. were cut into slices, the layers were removed, and the specimen was examined at X4, X10, X40, and X100 magnifications under light microscope. The sample was taken from the stem, which was located one to three centimeters from the tip. 14 different types of trichomes, including cylindrical, moniliform, simple uniseriate, non-glandular sessile, and palate types of glandular trichomes, are observed on the leaves, petiole, and stem of *Parthenium hysterophorus* L. These trichomes are primarily identified based on their structural differences. Trichomes are an important taxonomic tool for differentiating between species and genera. In respect to aridity, the study presents several new features that give future taxonomists a basic understanding of trichome diversity.

## Introduction

The family Asteraceae is one of the largest families of flowering plants, having a vast number of species that are distributed around the world and are significant economically (Stevens, 2008). According to Khaleel and Al-Dobaissi (2022), it has more than 1600 genera, over 25000 species, and is divided into 17 tibes and 3 subfamilies. The majority of Asteraceae species are shrubs, herbs, subshrubs, vines, and very infrequently, trees. The majority of its representatives are herbaceous with simple, frequently lobed leaves, capitulum-type inflorescences, and achenes-type fruits, which together with the pappus define its unit of dispersion (Roque & Bautista, 2008; Funk et al., 2009). The high number of species and their distribution in almost all habitats and vegetation formations make the Asteraceae family one of the most prosperous families (Beretta et al., 2008). This success is attributed to the family’s diverse secretory structure, specialized pollination and dispersal techniques, and phenotypic plasticity. According to Overbeck et al. (2015), the high representativeness of Asteraeae in send fields is likely due to these plants’ morphological and anatomical adaptations, which allow them to survive in harsh and constrained local environmental conditions like high temperatures and high luminosity, strong winds, low fertility soils, water deficit, and sandy soils. These adaptations, which are typically xeromorphic, include small leaves to decrease the surface area exposed to sunlight, the presence of secondary metabolites to defend against predation, and a significant number of trichomes on the vegetative organs (Boldrini, 2009). An interesting study that reveals the link as similarities or distinct properties of the many related species is the classification of family tribes based on their trichome characteristics (Kilian et al., 2009; Wagner et al., 2014).

*Parthenium hysterophorus* L., commonly known as Congress grass, is an annual or sporadic herb with many branches that belongs to the Asteraceae family. It is a member of the Heliantheae tribe and is notable for its potential harm to humans, animals, and the environment (Funk et al., 2009). The weed is thought to have migrated from North America to Australia and India, and in recent years it has become the seventh most hazardous weed in Africa, Asia, and Australia (Kaur et al., 2014). *Parthenium hysterophorus* L. grows abundantly in many places, including public lawns, forests, flood plains, agriculture, urban areas, overgrazed pastures, roadside, railway tracks, and residential plots. Although it may grow in most types of soil, weed is most common in clay loam and alkaline soils (Singh et al., 2004). *Parthenium hysterophorus* L. is used for composting and has been shown to have insecticidal, nematicidal, and herbicidal properties (Kumar et al., 2013).

The name trichome is applied to the epidermal outgrowth of various forms, structures, and functions (Esau, 1965). Taxonomic studies often find that the presence of a specific form of trichome can delimit species, genus, or family (Metcalfe and Chalk, 1950). Somehow, in phylogeny, the nature and structure of trichomes are highly significant. Carlquist (1976) defined more complex trichomes as advanced, while papilla, unicellular trichomes, and radially symmetrical trichomes are parallel to the leaf surface and are deemed as more primitive. According to Agren and Schemske (1994), they vary the boundary layer over the leaf surface, which functions as light plumbing after heat loss and serves to prevent water loss through transpiration. The glandular and non-glandular trichomes are the two main groups of trichomes, despite considering that their shape varies greatly (Sinha et al., 2001). Because glandular trichomes may synthesize, store, and exude secondary compounds that protect plants from insect predators and other biotic stresses Samsung smartphone (Ranger and Hower, 2001; Wagner et al., 2004; Ashfaq et al., 2019; Burnett et al., 1977). According to Bashir et al. (2020), it is one of the most significant methods used by modern taxonomists for the identification and differentiation of closely related taxa.

Many researchers, have stated that epidermal features are taxonomically significant (Metcalfe and Chalk, 1950; Shahzad et al., 2022; Manzoor et al., 2023). The present study deals with the diversity of trichomes for taxonomic purposes in *Parthenium hysterophorus* L. plant species from semi-arid regions of Barmer Rajasthan. Trichomes and their secretory products are known to function as defense mechanisms against herbivores (Lavin et al., 2001), according to Burnett et al. (1974), at least one sesquiterpene lactone is known to be an insect-feeding deterrent. Parthenin in *Parthenium hysterophorus* L. trichomes may also have the ability to inhibit feeding. Men who have allergic contact dermatitis develop parthenin deposits on exposed skin as a result of gland rupture (Rodriguez et al., 1976). Plant conservation is essential for future reference and taxonomic value. The way in which Nature Reserves and neighboring communities collaborate has a big influence on conservation management strategies as well as the local community (Guo et al., 2024). In order to conserve and better comprehend ecosystem services, it is also beneficial to raise education levels, create community norms and regulations, fortify human-nature relationships, and use ecological indigenous notions in environmental management (Ke et al., 2024). The major goals of the study are to examine and characterize the morphological forms and diversity of different trichomes that are present on the leaves, petioles, and stem of *Parthenium hysterophorus* L., which has been reported in semi-arid parts of the Indian Thar Desert.

## Materials and methods

### Study area

This research was carried out in the Semi-arid region of Barmer, Rajasthan, India. Rajasthan is located in the northwestern part of India, with the largest land area of 342,239 km^2^. Prime attractions of Rajasthan are its unique culture, Thar Desert, great forts, historical places, temples, and flora and fauna. Its western part has one of the major deserts of the world known as the “Great Indian Thar Desert”. Barmer is a district located in the western part of Rajasthan and forming a part of the Thar Desert and having one Desert National park. Natural vegetation in an arid climate is sparse and consists of perennial and ephemeral plants. The Desert area is characterized by xerophytic vegetation and is having a lot of Asteraceae plant species.

### Collection and identification of plant species

This study was conducted in Rajasthan’s semi-arid Barmer area. *Parthenium hysterophorus* L., a mature and healthy species of plant belonging to the Asteraceae family, was collected on regular field trips to different locations within the research area. Complete plants, including the root, stem, petioles, leaves, and flowers, were collected from research areas rocky spots, wasteland, and locations close to bodies of water. For epidermal analysis, the leaves, petiole, and stem sections were preserved in the FAA solution.

Plant specimens of *Parthenium hysterophorus L.* was identified using the “*Flora of Rajasthan*” (Shetty and Singh, 1987-1993), and “*Flora of Rajasthan East and Southeast Rajasthan*” (Tiagi and Aery, 2007) as well as other reference works. Plant specimen of *Genera Plantarum* Vol. I, II, and III, Bentham and Hooker, 1862-1883: Herbarium preparation and flora recording using Bentham and Hooker’s classification system. These plants have also been documented through photographs and Voucher specimens have been deposited at the herbarium of SRK Govt. College, Rajsamand, Rajasthan.

### Epidermal studies

An epidermal strip of healthy, mature leaves and stem was obtained. To create the epidermal peels on the abaxial and adaxial surfaces of the leaves as well as the stem, a tiny section was soaked in concentrated HNO3 in a petri dish for duration of roughly 6 to 12 hours. A pair of forceps was then used to move them into water in a petri dish. A sharp razor blade was used to delicately scrape off tiny slices of epidermis. Using a soft canal hair brush and water, the loose cells were removed from the epidermal peels until the desired epidermis underneath was reached. The epidermal peels were transferred to a clean glass slide, stained for five minutes in a 1% aqueous solution of safranin, and then mounted in 10% glycerol. The slides were inspected under a light microscope and given the proper labels. Slides were examined under a light microscope, and photos of trichomes were captured using a Samsung smartphone S20 FE 5G and a Sony Alpha digital camera with X4, X10, X40, and X100 objective lenses. The Trichome description was based on Inamdar et al. (1990) and Metcalf and Chalk (1979).

## Results

### Taxonomy

*Parthenium hysterophorus* L. Sp. Pl.988.1753; Raizada, Suppl. Duthie, Fl. Gangetic Plain 127.1976; Bhandari in Fl. Indian Desert 195.1978.

Synonym: *Argyrochaeta bipinnatifida* Cav., *Argyrochaeta parviflora* Cav., *Echetrosis pentasperma* Phil., *Parthenium glomeratum* Rollins, *Parthenium lobatum* Buckley, *Parthenium pinnatifidum* Stokes.

Taxonomic position: Campanulids (Dicotyledons), Asteraceae

Common Names: Carrot grass, Congress weed, Famine weed, *Parthenium* weed, Ragweed *Parthenium*.

Local name- Chatak Chadni

Flowering & fruiting- September-March

This exotic weed is generally spotted on bare lands, industrial areas, developing residential colonies, railway tracks, roads, drainage and around the ditch etc. Stem is cylindrical, solid and mature stems are greenish and covered with small soft hairs which are known as hirustle, stem become much harder as reach to maturity. The leaves are alternate arranged in two different forms. During the early stage it forms rosette habitat, later leaves are simple and deeply pinnatifid. Abaxial surface of leaves are covered with short, stiff hairs that lie close to the surface. Numerous small- flower head generally known as capitulum are organized in clusters at the top of the branches (in terminal penicles). Capitulum are off-white in color containing ray-florets. They also have various (15-60) small flowers (tubular florets) in the Centre surrounded by two rows of small green bracts (an involucre). Five small ‘seeds’ generally known as achenes are produced in each flower-head.

**Figure 1:**
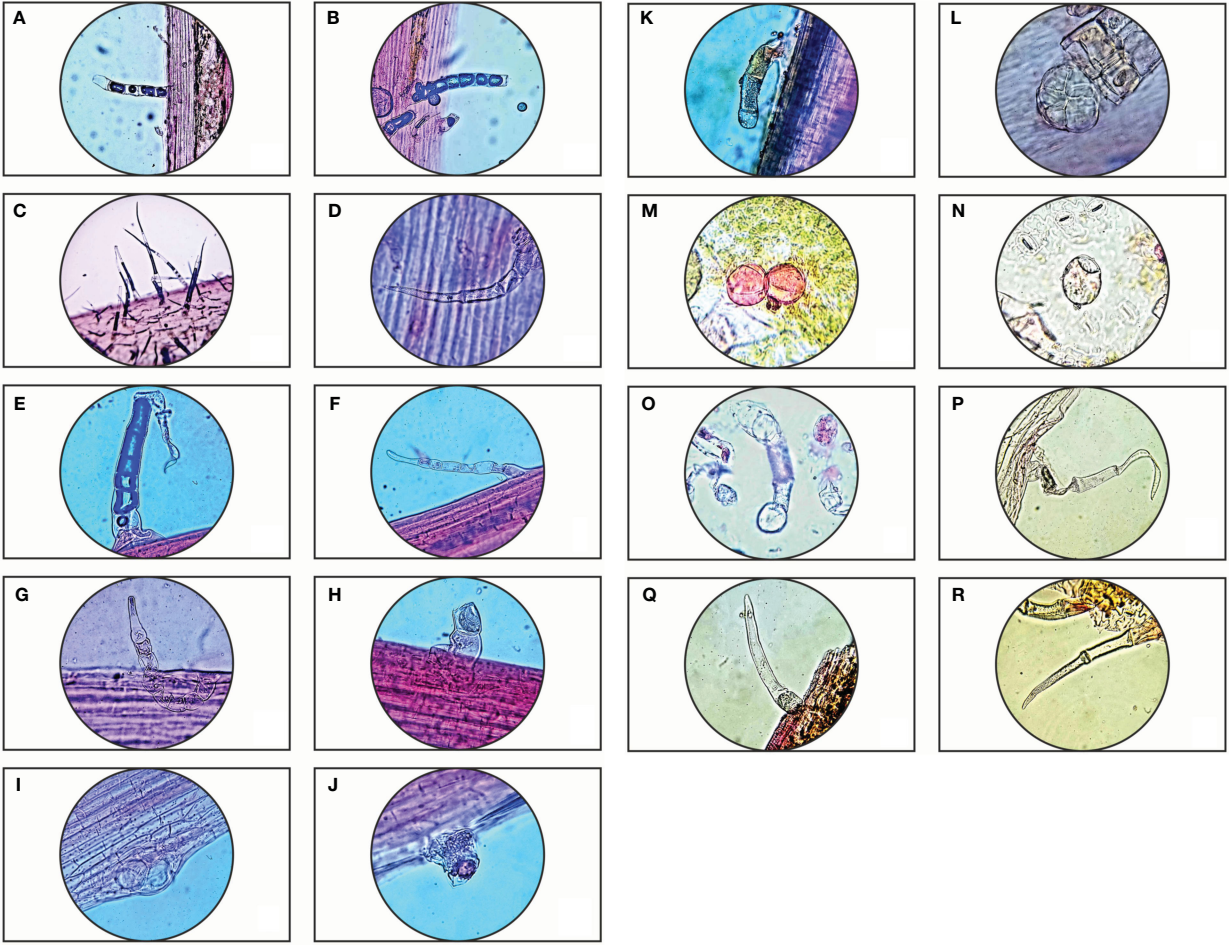
Trichome diversity of *Parthenium hysterophorus* L.: Stem- **(A, B)**; Leaf petiole surface- **(C–K)**; Abaxial & Adaxial surfaces of leaves- **(L–R)**.

**Figure 2:**
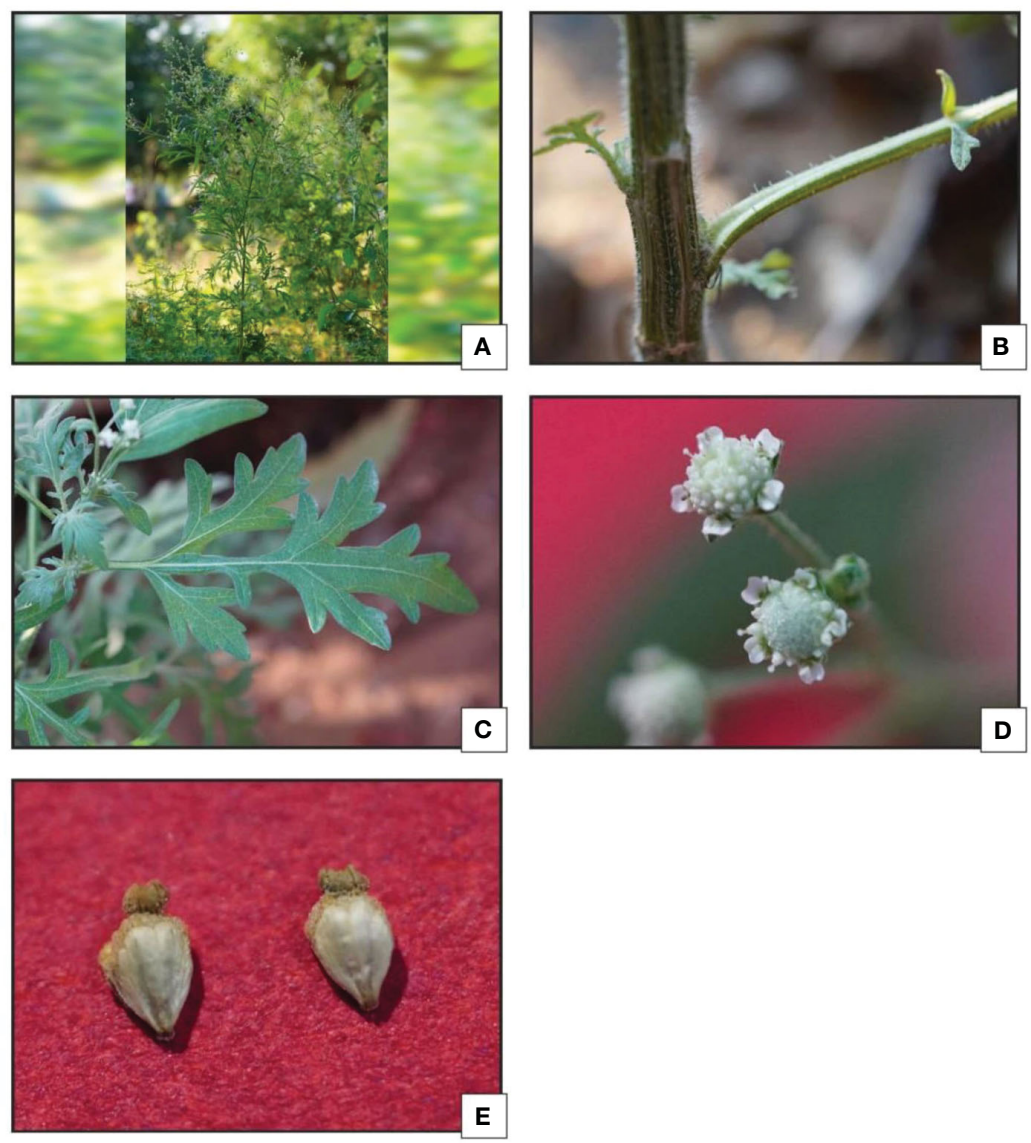
*Parthenium hysterophorus* L.: **(A, B)** Habit; **(C)** Leaves; **(D)** Inflorescence **(E)** Fruit achene.

*Parthenium hysterophorus* L. species was studied with consideration for the significance of foliar epidermal trichomes. **Table 1** lists the 14 distinct trichome types that were observed on the petioles, leaf surfaces and stem, while **Figures 1A–R**, **2A–E** displays a photomicrographs of the trichomes and morphology of *Parthenium hysterophorus* L.

**Table 1:** Morphological characteristics of Trichomes in *Parthenium hysterophorus* L.

Trichome position	Types of trichomes
**Stem**	1. Non-glandular trichome: Multicellular, uniseriate, blunt end (**Figures 1A, B**)
**Leaf petiole**	1. Non-glandular trichome: Uniseriate simple, multicellular, simple base, pointed end (**Figure 1C**)
	2. Non-glandular trichome: Moniliform, uniseriate, aseptate, simple base, pointed end (**Figure 1D**)
	3. Non-glandular trichome: Multicellular, long apical cell and filiform (**Figure 1E**)
	4. Non-glandular trichome: multicellular, oblong (**Figure 1F**)
	5. Glandular trichome: 9 celled, capitate head (**Figure 1G**)
	6. Glandular trichome: Sesile, capitate head (**Figures 1H–J**)
	7. Glandular trichome: Uniseriate, capitate head (**Figure 1K**)
**Abaxial & Adaxial surface of leaves**	1. Glandular trichome: Capitate head, multicellular-stalked (**Figure 1L**)
	2. Glandular trichome: Palate type, ball shaped, multicellular (**Figures 1M, N**)
	3. Glandular trichome: Capitate head, simple stalk, multicellular (**Figure 1O**)
	4. Non-glandular trichome: Uniseriate, multicellular, moniliform with long apical cell (**Figure 1P**)
	5. Non-glandular trichome: Uniseriate, aseptate, blunt end (**Figure 1Q**)
	6. Non-glandular trichome: Multicellular, long pointed tip (**Figure 1R**)

Trichomes are helpful in the systematic analysis of many taxa, according to research on trichomes in some plants of the family Asteraceae. The investigated taxa’s trichomes mostly fall into two categories: glandular and non-glandular. Non-glandular trichomes range from being unicellular to multicellular, whereas glandular trichomes are uniseriate, multicellular with capitate head and paltate type. Numerous researchers have documented the importance of trichomes at every level of species. The studied species, *Parthenium hysterophorus* L., displayed a higher density of trichomes to withstand the conditions of water stress because the study area is located in the dry semi-arid regions of Barmer, which means that it constantly faces water scarcity. As contrast to the adaxial surface, it is evident that the abaxial surface, leaf petiole, and stem surface have far higher trichome densities.

## Discussion

The presence of particular trichome types can be used in taxonomic analysis to designate species, genera, and families (Metcalf and Chalk, 1950). The ability of glandular trichomes to synthesize, store, and secrete secondary metabolites that aid in protecting plants from biotic stressors such as insect predation has received a lot of attention (Ranger and Hower, 2001). As an aspect of classification, Perveen et al. (2016) revealed that seventeen species belonging to the Asteraceae family exhibited trichomes that varied in size and shape. Trichomes are associated with xerophytic adaptation, but their forms are independent of environmental factors. Because each taxon exhibited distinct epidermal trichome characteristics that are utilized as indicators to distinguish between different taxa, the data recorded are useful in differentiating the study taxa*. Parthenium hysterophorus* L. leaves, petioles, and stem were found to have a variety of glandular and non-glandular trichome types.

In general, trichomes are thought to be among plants’ first lines of defense against abiotic stresses such UV radiation, water loss, and extreme temperatures (Ehleringer, 1982; Li et al., 2018; Oksanen, 2018). Trichomes have roles in defense as well as water usage techniques. They maintain the water content of leaves and regulate stomatal characteristics, among other things. According to Pan et al. (2021), epiphytic plants with trichomes had a greater percentage increase in leaf water content. Defense against herbivores is one of the major functions of trichomes, and both glandular and non-glandular trichomes have been well documented to deter herbivore movement and feeding (Kariyat et al., 2013; Kariyat et al., 2017; Kariyat et al., 2018; Kariyat et al., 2019; Ali et al., 2020). Morphology, density and dimensions relationships of subtypes of trichomes can be employed to find correlations between trichome characteristics with herbivore feeding intensity and behavior (Li et al., 2020).

In *Parthenium hysterophorus* L., Akhtar et al., 2022 also documented the existence of four different trichome types: shaggy, moniliform, simple multiseriate, and cylindrical. These trichomes are categorized based on their structural characteristics. The current investigation identified sixteen distinct trichome types: sessile glandular trichomes, non-glandular filiform trichomes, uniseriate trichomes with long and short apical cells, glandular trichomes with capitate heads, and palate ball-shaped trichomes. Akhtar et al. (2022) noticed that *Parthenium hysterophorus* L. exhibited well-developed trichomes based on its morphology and anatomy, which were reported in prior research.

## Conclusion

This investigation showed that there are differences in the type of trichomes at various taxonomic levels. Variations were noted at the same leaf’s abaxial and adaxial surfaces, as well as at the petiole, stem, and even the same surface. Thus, our work suggests that further thorough investigation is required for their further elaboration.

## Data availability statement

The raw data supporting the conclusions of this article will be made available by the authors, without undue reservation.

## Author contributions

V: Writing – original draft. DH: Supervision.
